# Endometrial Evaluation Using Video‐Assisted Hysteroscopy After Uterine Prolapse Management in a Mare

**DOI:** 10.1002/vms3.70903

**Published:** 2026-03-30

**Authors:** Gabriela Jaques Rodrigues, Francisco Décio de Oliveira Monteiro, Pedro Paulo Maia Teixeira

**Affiliations:** ^1^ Veterinary Medicine Institute Federal University of Pará Castanhal Pará Brazil; ^2^ Federal Institute of Tocantins (IFTO), Campus Araguatins Araguatins Tocantins Brazil

**Keywords:** Buhner's suture, endometrium, equine, evaluation of the reproductive tract, mare, uterine prolapse, video‐assisted hysteroscopy

## Abstract

This case report describes the successful application of video‐assisted hysteroscopy to evaluate endometrial recovery in a 6‐year‐old mare following treatment for complete uterine prolapse secondary to dystocia. Upon presentation, the uterus was manually reduced and supported with a Buhner's suture. A multimodal medical protocol, including broad‐spectrum antibiotics, anti‐inflammatory drugs and uterine lavage, was initiated. Five days after reduction, an endoscopic evaluation was performed using a dedicated videovaginoscope without uterine insufflation. The procedure provided high‐resolution imaging of the uterine lumen and revealed a grossly intact endometrial surface without visual evidence of haemorrhage, necrosis or purulent discharge, confirming the successful resolution of the initial trauma. The technique allowed for a thorough and comfortable examination, offering a definitive assessment of superficial mucosal integrity that exceeds the capabilities of transrectal ultrasonography. The mare was discharged on Day 7 with restored reproductive health and subsequently conceived, maintaining a pregnancy confirmed at a 6‐month follow‐up. This case underscores the efficacy of a combined medical and mechanical approach to the treatment of uterine prolapse. Furthermore, it highlights the significant diagnostic value of video‐assisted hysteroscopy as a valuable tool for post‐treatment monitoring, allowing direct visual confirmation of endometrial health and thus contributing to the preservation of fertility after a severe obstetric event. This approach may serve as a routine diagnostic tool in equine postpartum reproductive evaluation.

## Graphical abstract

Video‐assisted hysteroscopy for endometrial evaluation following uterine prolapse treatment in a mare. A 6‐year‐old mare presented with complete uterine prolapse after dystocia. Treatment included manual reduction, vulvar suture, antibiotics, anti‐inflammatories and uterine lavage. Five days post‐treatment, video‐assisted hysteroscopy using a dedicated videovaginoscope revealed intact endometrium without haemorrhage, necrosis or discharge. The mare recovered fully, conceived and maintained pregnancy at 6‐month follow‐up. This case highlights hysteroscopy as a superior tool for direct endometrial assessment post‐prolapse.

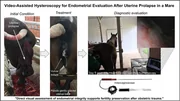

## 1 Introduction

Hysteroscopy in mares enables direct visual evaluation of the uterine lumen, endocervical canal and vaginal tract, allowing clinicians to assess reproductive health status and estrous cycle stages (Kohne et al. 2020; Abdelnaby et al. 2020). Hysteroscopy complements traditional transrectal palpation and ultrasonography by enabling real‐time evaluation of endometrial integrity and identification of subfertility causes (Eaton 2024). The results of reproductive tract assessment can be influenced by the quality, methodology and level of expertise of the examiner (Teixeira‐Soares et al. 2022).

Intraluminal examination of the reproductive tract is also valuable after conservative and/or surgical therapeutic interventions (Kutzler and Ing 2021). Evaluation of the reproductive tract after therapy, using follow‐up examinations such as ultrasonography and hysteroscopy, is crucial to monitoring reproductive recovery (Wilsher 2017). These exams are vital for detecting unresolved disorders that, if left untreated, may require specific interventions, from conservative medical management to surgical procedures like laser ablation of the uterine cyst. The lack of this diagnostic follow‐up allows such conditions to persist, often leading to subfertility or infertility in mares (Raudsepp 2020).

The incidence of severe pathologies such as uterine prolapse is significantly higher in herds lacking preventive measures. As demonstrated in this case, which was secondary to dystocia, preventive measures are crucial and include diligent supervision during foaling for timely intervention, nutritional management to prevent hypocalcemia and obesity and protocols to minimise prolonged strain (Barba et al. 2020). Rare conditions, such as uterine prolapse, a medical emergency compromising mare health and fertility, can also arise (Boye et al. 2022).

Uterine prolapse is a rare but serious medical emergency that severely compromises mare health and fertility. Its incidence is higher in peripartum mares that are unobserved or lack adequate supervision, as the underlying causes, such as dystocia and prolonged straining, often go unrecognised and unassisted, leading to complications (Boye et al. 2022; Barba et al. 2020).

Direct luminal inspection is critical after uterine prolapse to evaluate post‐treatment recovery and rule out severe complications. Without endoscopic visualization, subtle but significant sequelae such as focal endometrial necrosis, adhesions (synechiae), persistent haemorrhagic areas or subclinical endometritis may remain undetected, potentially leading to chronic infection, fibrosis and permanent infertility. Therefore, this report describes the use of video‐assisted hysteroscopy in a mare after uterine prolapse treatment to provide a definitive assessment of endometrial health.

## 2 Case

**FIGURE 1:**
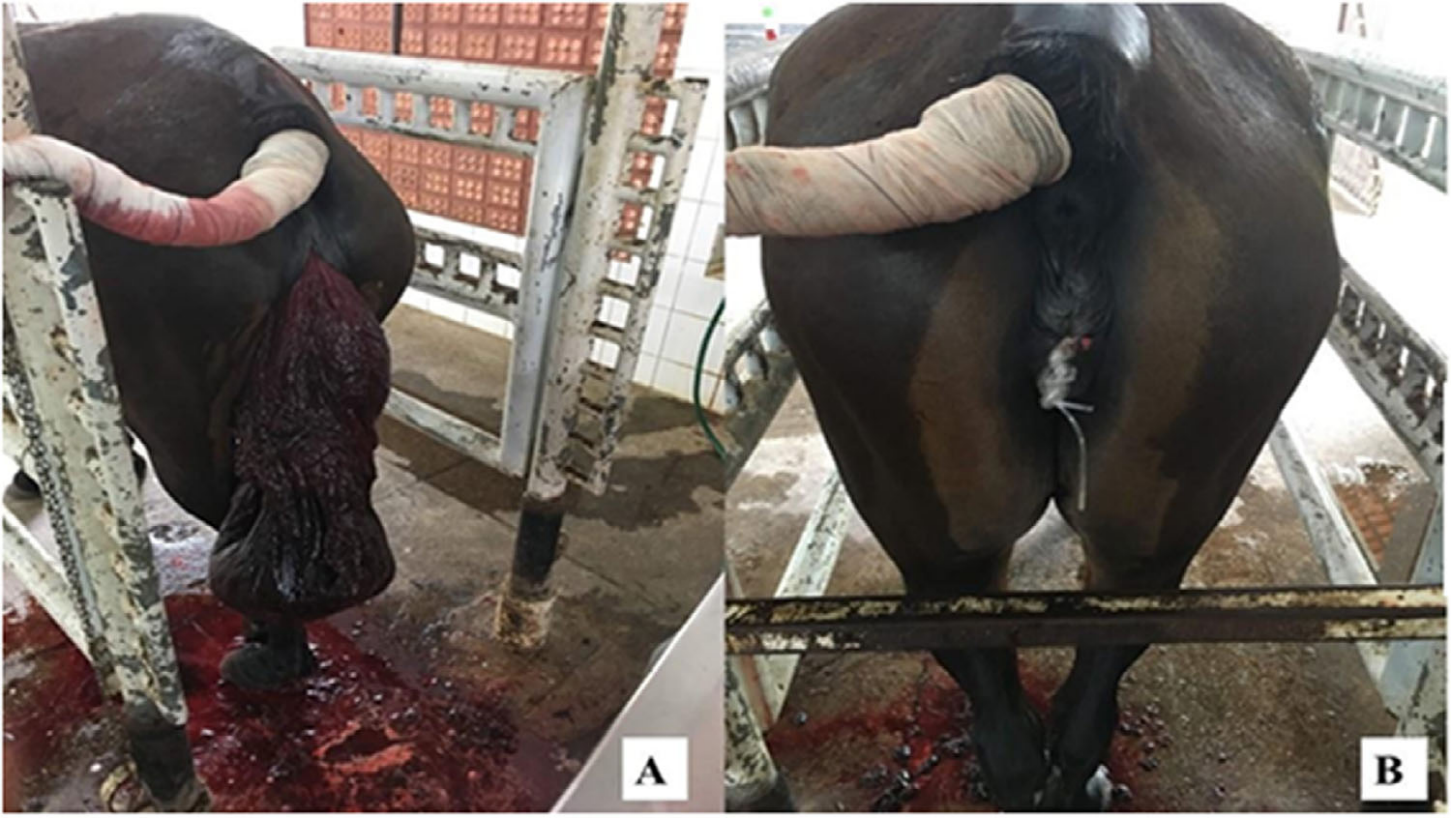
Mare with uterine prolapse and after treatment. (A) Mare with uterine prolapse. (B) Vulva with a Buhner's suture applied to prevent recurrence of prolapse. Although the suture pattern may appear altered, its primary function is to provide adequate vulvar closure and support after manual reduction of the uterus.

Video‐assisted hysteroscopy was performed 5 days after treatment of a mare with uterine prolapse (Animal Use Ethics Committee—CEUA/Federal University of Pará—UFPA no. 8762300120). The mare was 6 years old, weighed 456 kg and was of the Manga‐Larga Marchador breed. This mare was referred to the Pará Federal University Veterinary Hospital (Large Animal Veterinary Hospital—HOVET/UFPA) following an unobserved abortion at 10 months of gestation approximately 12 h prior. On the basis of the owner's report of prolonged and difficult delivery, the event was characterised as dystocia. The owner reported that the mare had been found with the uterus prolapsed shortly after delivery and had not received treatment prior to referral. The main complaint upon arrival was the presence of complete uterine prolapse (Figure 1A).

Upon initial presentation, the mare was bright, alert and responsive. Physical examination revealed moist pink mucous membranes with a capillary refill time of <2 s, palpable normal peripheral lymph nodes and a body temperature of 36°C. The mare exhibited intermittent, mild straining but did not show other signs of significant distress. The owner confirmed that the placenta had been intact approximately 1 h after the unobserved abortion, which immediately preceded the discovery of the prolapse. A blood sample was collected aseptically from the jugular vein using a 20‐gauge sterile needle and syringe then transferred to EDTA tubes for haematological analysis.

The blood count revealed hypochromic, macrocytic and regenerative anaemia, consistent with acute blood loss. On the basis of the PCV of 15% (reference range 24%–44%), the estimated blood loss was approximately 18–20 L. Despite this significant anaemia, the mare's cardiovascular compensation was evident as her mucous membranes remained pink and moist with a capillary refill time <2 s, and she was bright, alert and responsive. The leukogram showed no significant changes, indicating the absence of a marked inflammatory response at the time of sampling (Tables 1 and 2).

**TABLE 1:** Haematological results of the complete blood count (CBC) of the patient.

Parameter	Result	Reference range
Red blood cells (10^6^/mm^3^)	1.8	5.5–9.5
Haemoglobin (g/dL)	5	8.0–14.0
Packed cell volume (%)	15	24–44
MCV (fL)	83.3	39–52
MCHC (%)	33.3	31–35
Total plasma protein (g/dL)	7.2	5.8–8.7
Fibrinogen (mg/dL)	400	100–400

Abbreviations: MCHC, mean corpuscular haemoglobin concentration; MCV, mean corpuscular volume.

**TABLE 2:** Leukogram of the patient with the results.

Leukogram				
Differential count	%	Cells/µL	%	Cells/µL
Total leukocytes	6.7	5.2–13 (×10^3^/mL)		
Monocytes	8	536	2–10	120–1200
Lymphocytes	36	2412	15–50	500–6000
Eosinophils	2	134	2–12	120–1440
Band cells	—	—	0–2	0–240
Segmented neutrophils	54	3618	35–75	2100–9000
Basophils	—	—	Rare	0–360
Metamyelocytes	—	—	Rare	Rare

Abbreviations: MCHC, mean corpuscular haemoglobin concentration; MCV, mean corpuscular volume; TPP, total plasma protein.

The complete blood count (CBC) indicated significant acute blood loss, characterised by severe hypochromic, macrocytic regenerative anaemia. Key findings included a profoundly low packed cell volume (PCV: 15%; reference range: 24%–44%), low red blood cell count (1.8 × 10^6^/µL; ref.: 5.5–9.5) and low haemoglobin concentration (5 g/dL; ref.: 8.0–14.0). The leukogram was unremarkable, with all values falling within normal reference intervals, suggesting no active inflammatory process.

The patient was immediately prepared for the uterine prolapse correction procedure. The mare was sedated with xylazine (0.5 mg/kg intravenous) to facilitate handling and reduce strain. Then, a low caudal epidural block was administered using lidocaine (2%; 0.2 mg/kg) in the first intercoccygeal (Co1–Co2) space to provide analgesia and further control tenesmus. Hyoscine butylbromide (0.3 mg/kg intravenous) was administered to relax the uterine smooth muscle and facilitate replacement. Following perianal and vulvar asepsis, the uterus was carefully inspected; it was edematous but without visible signs of rupture or severe trauma.

The replacement was performed manually over a period of approximately 15 min using large amounts of lubricating gel to minimise further tissue damage. Once the uterus was fully reduced, a Buhner's suture was placed on the vulva to provide support and prevent recurrence (Figure 1B). Antitetanus serum was administered in a single dose and for 5 days of drug treatment based on streptomycin (Pentabiotic) at a dose of 22,000 IU/kg (intramuscular/once daily), meloxicam 2% (Maxicam) at a dose of 0.6 mg/kg (intramuscular/once daily), multivitamin supplement (Hemolitan) at 20 mL/animal (orally/once daily) and uterine lavage with 0.5% iodine solution (once daily). On the last day, phenylbutazone (Phenylbutazone) was applied at a dose of 2.2 mg/kg (intravenous/twice daily).

**FIGURE 2:**
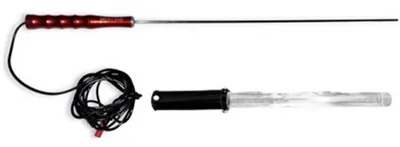
Video vaginoscope Scope VOR&GDI.

**FIGURE 3:**
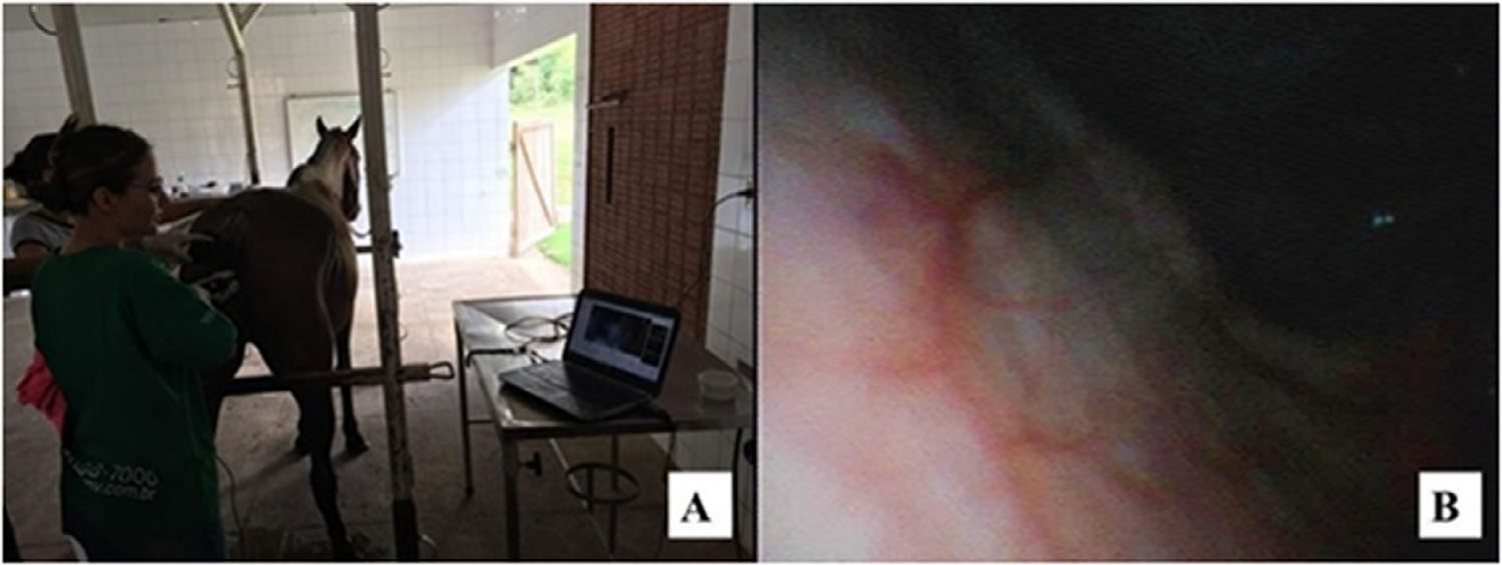
Performing video‐assisted hysteroscopy in mare and viewing the endometrium. (A) Performing video‐assisted hysteroscopy in mare and viewing the endometrium. (B) View of the uterine body obtained without insufflation, demonstrating a well‐preserved endometrial mucosa. Despite the lack of distension, which can limit panoramic visibility compared to an insufflated uterus, the mucosal architecture appears intact with no evidence of haemorrhage, necrosis or inflammation after treatment.

To evaluate endometrial recovery during the treatment protocol, the patient underwent hysteroscopy 5 days after initial uterine replacement and the start of medical therapy. This timing was selected to allow for initial mucosal healing and reduction of acute oedema and inflammation, while still enabling early detection of potential complications such as necrosis or infection before the completion of the full antimicrobial course. Performing the procedure prior to the cessation of NSAIDs and antibiotics also ensured patient comfort and minimised procedural discomfort in the recently traumatised uterus. The examination was performed using the Scope VOR&GDI Videovaginoscope4, which is made up of a tubular acrylic speculum measuring 3.5 cm in diameter and 32 cm in length (washable, light and transparent) and a Scope 160K optic with a microcamera and a LED on the rod (Figure 2). The speculum was introduced into the mare's vagina and advanced through the cervical canal to access the uterine body (Figure 3A).

Hysteroscopic evaluation confirmed complete endometrial recovery. The mucosal surface was smooth and continuous, with the absence of haemorrhagic lesions, necrotic tissue, oedema or purulent discharge, indicating the resolution of the previous prolapse event (Figure 3B). The mare was discharged 7 days after treatment with the uterus correctly positioned in the pelvic cavity and no clinical signs of discomfort. At 6‐months of follow‐up, the mare was confirmed to have conceived and maintained a pregnancy, demonstrating the efficacy of the treatment in restoring reproductive functionality.

## 3 Discussion

The decision to utilization Buhner's suture in this case, while indeed less common in equine practice than a vulvoplasty, was based on the specific clinical requirements presented (Schumacher and O'Brien 2021a). The mare exhibited persistent, though mild, strain after manual reduction of significant prolapse. In such acute scenarios, where the primary concern is immediate mechanical recurrence due to increased intra‐abdominal pressure and compromised pelvic support structures, Buhner's suture provides substantial circumferential support that a standard or modified Caslick's procedure cannot offer (Chisholm et al. 2021; Dascanio 2021). The use of a Buhner's suture for mechanical support following uterine reduction has been reported in similar cases, where it effectively prevented recurrence and supported healing in the acute postpartum period (Farjanikish et al. 2017).

The Caslick's operation is highly effective for long‐term management of pneumovagina by opposing the dorsal vulvar labia but is not designed to counteract the forceful straining associated with post‐prolapse recovery or profound tenesmus (Sarath et al. 2025). Therefore, Buhner's technique was selected as a pragmatic intervention to provide critical external support during the initial healing phase, preventing re‐prolapse and allowing underlying tissues to regain tone. This approach, combined with epidural analgesia, created a multi‐modal strategy to manage tenesmus and protect reduction until the mare's natural supportive structures could recover, ultimately contributing to the successful outcome documented by hysteroscopy.

Video‐assisted hysteroscopy in the mare after uterine prolapse treatment proved to be effective, allowing a detailed luminal inspection of the vagina and uterus. The examination was performed without uterine insufflation to minimise iatrogenic discomfort and the risk of trauma or inflammation in the recently prolapsed organ. The slender, atraumatic and flexible acrylic speculum of the device, combined with the standing sedation of the mare and the operator's careful manual advancement, allowed safe navigation through the cervical canal and the uterine body and horns. Although the characteristic appearance of the endometrial mucosa in both uterine horns could be thoroughly evaluated, visualization of the oviductal papillae was not achieved due to the anatomical constraints of the equine uterotubal junction and the conscious state of the patient, which is a recognised limitation of standard hysteroscopy. This technique allows clinicians to directly identify reproductive pathologies that affect fertility and monitor treatment responses, as demonstrated in this case (Kutzler and Ing 2021; Teixeira‐Soares et al. 2022).

The endoscopic device provided high‐quality imaging of the uterine lumen, with adequate sharpness and magnification. Optimal cavity illumination and high‐resolution optics are critical for diagnostic accuracy in endosurgery (Alvarez et al. 2021; He et al. 2021). The video‐assisted hysteroscopy can make procedures such as artificial insemination, biopsies, uterine lavage and other diagnostic or therapeutic interventions easier, even more so when instruments have designs that include ports for biopsy needles, lavage pipettes and insemination applicators (Teixeira‐Soares et al. 2022; Husby et al. 2019).

The anatomical compatibility of the device and its components allowed complete mucosal examination without causing discomfort to the mare post‐prolapse reduction. Notably, diagnostic hysteroscopy carries a risk of iatrogenic endometrial contamination, as evidenced by a study in which 50% of mares developed pathogenic microbial contamination post‐procedure, underscoring the importance of aseptic technique and potential post‐procedural care (Schiemann et al. 2001).

The operator benefited from improved ergonomics, performing the examination from an upright position while viewing high‐resolution real‐time images on a stationary monitor. This was facilitated by the videovaginoscope's design, which features a lightweight, tubular acrylic speculum that was introduced into the vaginal vault and guided through the cervical canal to access the uterine body, allowing for a thorough evaluation without the need to assume strenuous positions (Easley et al. 2017; He et al. 2021).

Uterine prolapse is rare in mares due to the anatomical support of the reproductive tract. In this case, the prolapse originated from vaginal inversion secondary to the forceful uterine contractions associated with dystocia described at presentation (Alamaary and Ali 2020; Lu and Sprayberry 2021). Immediate veterinary intervention was critical, as complications such as uterine rupture, ligament damage or endotoxemia worsen the prognosis (Lu and Sprayberry 2021; Schumacher and O'Brien 2021b). Uterine prolapse remains a rare but serious postpartum emergency, often associated with dystocia, retained placenta or abortion, necessitating prompt intervention to manage contamination, injury, and tenesmus (Paidar Ardakani 2025).

The treatment protocol was designed to address the primary sequelae of uterine prolapse: contamination, inflammation, pain and fluid loss. Uterine repositioning was the immediate priority, facilitated by epidural analgesia. Broad‐spectrum antimicrobial therapy was initiated using streptomycin (Pentabiotic), a drug that contains dihydrostreptomycin along with penicillin and other antibiotics, providing coverage against a wide range of potential gram‐negative and anaerobic contaminants acquired from environmental exposure during prolapse (Dunkel and Johns 2015; Tallon et al. 2023). Anti‐inflammatory therapy began with meloxicam for its potent anti‐cyclooxygenase‐2 (COX‐2) activity and favourable safety profile for multi‐day use, aiming to control systemic inflammation and pain (Lemonnier et al. 2022).

On the last day, therapy was switched to phenylbutazone due to its more potent analgesic effect, ensuring patient comfort during the transition to discharge. Once‐daily uterine lavage with a 0.5% iodine solution was deemed sufficient based on the mare's rapid clinical improvement; the absence of purulent discharge on subsequent evaluations and the excellent endoscopic appearance of the endometrium after 5 days confirmed the efficacy of this frequency in resolving contamination without risking iatrogenic irritation from more frequent flushing. This multi‐modal approach effectively managed the risks of metritis, endotoxemia and adhesions.

Post‐treatment hysteroscopy confirmed endometrial integrity, with no evidence of haemorrhage, necrosis or discharge. Endoscopic evaluation provided a critical advantage over transrectal ultrasonography, allowing direct luminal visualization and definitive diagnosis of superficial endometrial integrity. Unlike ultrasonography, which excels in evaluating mural architecture and fluid accumulation but can miss subtle mucosal lesions, hysteroscopy allowed the direct identification of normal vascular patterns and the unambiguous ruling out of complications such as adherent fibrinous strands, microhaemorrhages or focal necrosis that might not be sonographically apparent (Alvarez et al. 2021; Teixeira‐Soares et al. 2022).

This application of a dedicated video vaginoscope represents a practical advancement within the well‐established field of equine reproductive endoscopy. Although hysteroscopy is a common diagnostic tool in referral settings, the integrated design of this device offers a streamlined alternative to conventional systems. The findings related to patient comfort align with those of Rodrigues et al. (2023) in cattle, where a similar videoscopic approach also provided high‐quality mucosa imaging with minimal discomfort, enhancing diagnostic precision for post‐prolapse evaluation (Kutzler and Ing 2021; Teixeira‐Soares et al. 2022).

In the new video assisted method, high‐resolution optics and integrated illumination, which Rodrigues et al. (2023) highlighted as pivotal for bovine vaginoscopy, were equally transformative in equine hysteroscopy. The video‐assisted hysteroscopy makes it possible to detect subtle endometrial lesions (e.g., haemorrhage or necrosis) post‐prolapse mirrors its success in identifying bovine vaginal pathologies, such as mucosal haemorrhagic points and cervical mucus plugs (Rodrigues et al. 2023). This underscores its applicability to cross‐species for luminal diagnostics (Alvarez et al. 2021).

Although video‐assisted hysteroscopy is previously described for procedures such as endometrial cyst ablation in mares, the novel aspect of the present technique is the use of a dedicated videovaginoscope. This specific device integrates a high‐resolution microcamera directly onto a lightweight tubular acrylic speculum, eliminating the need for a separate endoscopic tower and potentially offering a more streamlined and portable approach for intrauterine evaluation and therapeutic interventions compared to conventional hysteroscopic systems. Rodrigues et al. (2023) reported that 23% of heifers exhibited discomfort with traditional speculums, whereas video‐assisted hysteroscopy caused none. The minimally invasive approach could revolutionise routine equine gynecologic exams, particularly for post‐partum or high‐risk cases (Lu and Sprayberry 2021).

As a single‐case report, the findings presented here, while compelling, are inherently limited in their generalizability. The successful outcome cannot be definitively attributed solely to any single component of the protocol described. Furthermore, the optimal timing for post‐reduction hysteroscopy or the comparative efficacy of different medical regimens requires validation through controlled studies involving larger case series.

## 4 Outcome

The patient showed a highly positive response to the multi‐modal treatment protocol. Hysteroscopic evaluation 5 days after prolapse reduction, performed using the Scope VOR&GDI Videovaginoscope, revealed a fully recovered endometrium. The examination revealed no evidence of haemorrhage, necrosis, inflammation or purulent discharge (Figure 3B), confirming successful resolution of the initial trauma and contamination. The mare was subsequently discharged on Day 7 with the uterus correctly positioned and showing no clinical signs of discomfort. A 6‐month follow‐up confirmed complete restoration of reproductive function, as the mare had conceived and successfully maintained a pregnancy.

## 5 Conclusion

This case demonstrates the successful management of uterine prolapse in a mare through manual reduction, supported by a Buhner's suture and a targeted medical protocol. Furthermore, it highlights the significant diagnostic value of video‐assisted hysteroscopy for post‐treatment evaluation. The technique provided a definitive and direct evaluation of endometrial health, superior to ultrasonography for visualizing mucosal integrity, and confirmed the efficacy of the intervention without causing discomfort to the patient. The positive long‐term result of breeding underscores that this combined approach, prompt medical and mechanical treatment followed by endoscopic evaluation, may be effective in preserving fertility after a severe obstetric event.

## Author Contributions

Gabriela Jaques Rodrigues, Francisco Décio de Oliveira Monteiro and Pedro Paulo Maia Teixeira were responsible for the diagnosis and treatment of the patient, conception and design of the manuscript. Gabriela Jaques Rodrigues and Francisco Décio de Oliveira Monteiro were responsible for the collection, analysis and systematization of data with article writing and critical review. Gabriela Jaques Rodrigues, Francisco Décio de Oliveira Monteiro and Pedro Paulo Maia Teixeira were responsible for analysing and systematizing data and images, as well as critically reviewing the writing of the manuscript. All authors actively contributed to the manuscript and approved the final version to be published.

## Funding

The authors have nothing to report.

## Conflicts of Interest

The authors declare no conflicts of interest.

## Manufacturers’ Addresses

^1^ Zoetis, Anápolis, GO, Brazil.

^2^ Ourofino, SP, Brazil.

^3^ Vetnil, SP, Brazil.

^4^ Gold Diagnóstic Instruments (GDI), SP, Brazil.

## Data Availability

The data that support the findings of this study are available from the corresponding author upon reasonable request.
